# Supplementary material for: Spontaneous Physical Activity in Obese Condition Favours Antitumour Immunity Leading to Decreased Tumour Growth in a Syngeneic Mouse Model of Carcinogenesis
Source: Cancers (Basel). 2021 Dec 23;14(1):59. doi: 10.3390/cancers14010059 (PMC8750291; doi:10.3390/cancers14010059)
Supplement: Supplementary file 1 [file cancers-14-00059-s001.zip › cancers-1462773-supplementary.pdf]

Table S1. Composition of high-fat diet.

Composition	High-fat diet
Total energy content (kcal/g)	4.1
Carbohydrates (%)	39
Fat (%)	45
Proteins (%)	16
Sucrose (mg/g)	220
ω6 (mg/g)	23.2
ω 3 (mg/g)	3.8
Ratio ω6/ω3	6.2
Mineral mixture[1] (AIN-93 M) (mg/g)	50
Vitamin mixture[2] (AIN-93 M) (mg/g)	10

Values for each macronutrient are expressed in percentage of total energy content and ingredients in milligrams per gram of diet. [1]AIN-93 M mineral mixture (mg/g): AIN-76 mineral mixture (grams/kg): calcium phosphate 500, sodium. chloride 74, potassium citrate 2220, potassium sulfate 52, magnesium oxide 24, manganous carbonate 3.5, ferric citrate 6, zinc carbonate 1.6, cupric carbonate 0.3, potassium iodate 0.01, sodium selenite 0.01, chromium potassium sulfate 0.55, sucrose 118.03. [2]AIN-93 M vitamin mixture (mg/g): thiamin HCl 0.6, riboflavin 0.6, pyridoxine HCl 0.7, niacin 3, calcium pantothenate 1.6, folic acid 0.2, biotin 0.02, vitamin B12 1, vitamin A (500,000 U/gm) 0.8, vitamin D3(400,000 U/gm) 0.25, vitamin E acetate (500 U/gm) 10, menadione sodium bisulfite 0.08, sucrose 981.15.

Table S**2.** Antibodies used for flow cytometry staining.

Antibody	Fluorochrom	Clone	Provider
**Extracellular staining**			
CD3ε	Biotin	145-2C11	MACS Miltenyi
CD4	AF700	GK1.5	eBioscience
CD4	PE	GK1.5	MACS Miltenyi
CD8α	FITC	53-6.7	MACS Miltenyi
CD11b	PerCP Cy5.5	M1/70.15	eBioscience
CD11c	AF700	N418	eBioscience
CD19	FITC	6D5	MACS Miltenyi
CD25	PE	PC61.5	eBioscience
CD45R (B220)	PerCP	RA3-6B2	MACS Miltenyi
CD49b (DX5)	PE	DX5	MACS Miltenyi
CD119	PE	REA189	MACS Miltenyi
CD120a	PE	REA252	MACS Miltenyi
CD124	PE	REA235	MACS Miltenyi
CD138	Biotin	REA104	MACS Miltenyi
CD184 (CXCR4)	PE	REA107	MACS Miltenyi
CD204	Biotin	REA148	MACS Miltenyi
F4/80	Pe	REA126	MACS Miltenyi
Ly6C	PE	REA287	MACS Miltenyi
Ly6G	PerCPVio700	REA526	MACS Miltenyi
MHC II	PE	REA528	MACS Miltenyi
NK1.1	PerCPVio700	PK136	MACS Miltenyi
Streptavidin	PeCy7	Streptavidin	eBioscience
Streptavidin	FITC	Bio3-18E7	MACS Miltenyi
**Intracellular staining**			
FoxP3	Biotin	FJK-16s	eBioscience

Table S3. PCR primers.

Gene name	Accession number (NCBI)	Primer sequences	Direction	Amplicon size
Gapdh	NM_001289726.1	5′-ACCCCAGCAAGGACACTGAGCAAG-3′	Forward	109 bp
		5′-GGCCCCTCCTGTTATTATGGGGGT-3′	Reverse	
Tbx21	NM_019507.2	5′-CACTAAGCAAGGACGGCGAA-3′	Forward	72 bp
		5′-TAATGGCTTGTGGGCTCCAG-3′	Reverse	
Gata3	NM_008091.3	5′-CTCCTTGCTACTCAGGTGATCG-3′	Forward	92 bp
		5′-AGGGAGAGAGGAATCCGAGT-3′	Reverse	
Cd8α	NM_009857.1	5′-AAGGGGACCGGATTGGACTT-3′	Forward	97 bp
		5′-CTAGCGGCCCTTGGGACATTTG-3′	Reverse	
DX5	NM_008396,2	5′-TGCGGCTGCTAATGCTAGTT-3′	Forward	150 bp
		5′-CCAGTAGCCAGTTGCCTTGT-3′	Reverse	
CD1d	NM_007639.3	5′-CCTTTGTGTACCAGTCCGGG-3′	Forward	144 bp
		5′-TTTTGCTGGGCTTCAGATTGTC-3′	Reverse	
Granzyme A	NM_010370.2	5′-TCATTGGAGGAGACACGGTT-3′	Forward	170 bp
		5′-TGATTGAGTGAGCCCCAAGA-3′	Reverse	
Granzyme B	NM_013542	5′-TCGACCCTACATGGCCTTAC-3′	Forward	389 bp
		5′-CACACTCCCGATCCTTCTGT-3′	Reverse	
Perforin 1	NM_011073	5′-GATGTGAACCCTAGGCCAGA-3′	Forward	200 bp
		5′-TGGTAAGCATGCTCTGTGGA-3′	Reverse	

Table S4. Cytokines and matrix metalloproteases.

**group**	**Standard**				**Enriched**			
Tissue	plasma	IAT	Gas.	tumour	plasma	IAT	Gas.	tumour
VEGF-A	ND	0.0144 ± 0.0164	0.069 ± 0.081	0.578 ± 0.380	ND	0.057 ± 0.052	0.238 ± 0.045	0.620 ± 0.328
G-CSF	159383 ± 75155	48.43 ± 54.67	10.43 ± 10.66	218.9 ± 112.8	791200 ± 617840	35.95 ± 26.32	24.72 ± 16.47	326.5 ± 152.5
IFN-γ	ND	0.11 ± 0.17	0.44 ± 0.26	0.63 ± 0.47	ND	0.09 ± 0.07	0.69 ± 0.31	0.58 ± 0.53
IL-1 α	186.6 ± 304.5	3.28 ± 2.32	5.38 ± 1.21	1.34 ± 1.21	328.8 ± 493.1	3.23 ± 2.26	11.26 ± 2.25	1.42 ± 1.32
IL-4	10.7 ± 6.1	0.008 ± 0.004	0.012 ± 0.004	0.010 ± 0.004	70.6 ± 102.5	0.004 ± 0.003	0.013 ± 0.012	0.005 ± 0.002
IL-6	0.13 ± 0.10	1.41 ± 0.81	1.61 ± 0.56	2.11 ± 0.52	0.05 ± 0.09	1.10 ± 0.39	1.96 ± 0.85	2.05 ± 1.30
IL-10	0.001 ± 0.001	0.147 ± 0.121	1.52 ± 0.92	0.241 ± 0.190	27.4 ± 47.6	0.172 ± 0.140	3.37 ± 1.16	0.164 ± 0.109
IL-15	226.5 ± 36.5	0.39 ± 0.11	1.29 ± 0.19	0.28 ± 0.14	0.001 ± 0.001	0.001 ± 0.001	0.32 ± 0.48	0.001 ± 0.001
IP-10	12692 ± 13406	0.129 ± 0.088	0.46 ± 0.16	20.14 ± 8.63	14010 ± 10265	0.095 ± 0.032	0.86 ± 0.24	14.59 ± 5.03
MIG	5356.7 ± 1903.3	0.040 ± 0.044	0.001 ± 0.001	18.03 ± 4.74	4016.3 ± 1636.3	0.108 ± 0.088	0.062 ± 0.099	17.69 ± 7.57
LIF	6.37 ± 1.79	0.020 ± 0.013	0.006 ± 0.001	0.632 ± 0.274	0.001 ± 0.001	0.001 ± 0.001	0.001 ± 0.001	0.671 ± 0.216
MIP-1α	30.7 ± 10.7	ND	ND	3.62 ± 2.39	30.7 ± 2.6	ND	ND	2.49 ± 0.28
RANTES	4.35 ± 7.25	0.008 ± 0.011	0.003 ± 0.005	1.28 ± 0.47	0.67 ± 1.17	0.018 ± 0.021	0.040 ± 0.019	1.21 ± 0.48
MCP-1	166.7 ± 277.8	0.018 ± 0.030	0.001 ± 0.001	21.24 ± 8.52	636.3 ± 954.4	0.193 ± 0.155	0.441 ± 0.705	27.26 ± 7.02
TNF-α	ND	0.030 ± 0.020	0.008 ± 0.014	0.132 ± 0.027	ND	0.078 ± 0.030	0.116 ± 0.122	0.134 ± 0.039
PAI-1	182.5 ± 96.6	10.38 ± 4.29	2.09 ± 0.47	135.2 ± 46.6	140.2 ± 120.4	6.74 ± 3.28	2.48 ± 0.99	165.9 ± 85.3
Resistin	36.0 ± 11.5	18.2 ± 9.9	3.46 ± 0.59	5.12 ± 3.07	30.8 ± 13.1	17.9 ± 4.9	4.05 ± 0.64	3.49 ± 1.59
Leptin	41.3 ± 25.0	5.20 ± 1.35	0.46 ± 0.21	0.79 ± 0.32	76.0 ± 74.5	4.62 ± 2.37	0.09 ± 0.06	0.41 ± 0.23
Adiponectin	3829.9 ± 355.8	3010.7 ± 564.6	3554.9 ± 244.7	1146.7 ± 376.3	4599.9 ± 1205.4	2902.4 ± 758.7	3565.5 ± 516.6	1032.9 ± 247.2
Myostatin	9040 ± 4430	0.001 ± 0.001	0.001 ± 0.001	0.001 ± 0.001	0.001 ± 0.001	0.001 ± 0.001	0.001 ± 0.001	0.001 ± 0.001
Irisin	37.9 ± 20.7	1.09 ± 0.47	3.02 ± 0.56	0.14 ± 0.09	16.5 ± 2.5	1.69 ± 1.04	4.75 ± 1.98	0.29 ± 0.15
MMP2	ND	184.7 ± 65.3	101.7 ± 14.8	610.0 ± 188.7	ND	122.5 ± 35.0	52.3 ± 15.3	364.4 ± 73.7
MMP3	ND	267.6 ± 177.4	40.6 ± 11.3	1484.7 ± 1366.2	ND	146.9 ± 39.4	44.3 ± 12.4	273.7 ± 13.5
proMMP9	ND	479.6 ± 350.7	108.1 ± 29.4	17239 ± 14104	ND	179.7 ± 103.3	80.3 ± 16.2	5934 ± 1921
MMP12	ND	36.0 ± 29.2	0.001 ± 0.001	96.7 ± 57.7	ND	21.8 ± 9.4	0.001 ± 0.001	64.8 ± 20.8

The cytokine and matrix metalloproteases secretions in plasma and tissues. Results are in pg/mg of proteins and mean ± SEM (*n* = 5/group). ND: non determined. IAT: Ingunal adipose tissue; Gas.: gastrocnemius muscle.

T cytotoxic lymphocytes

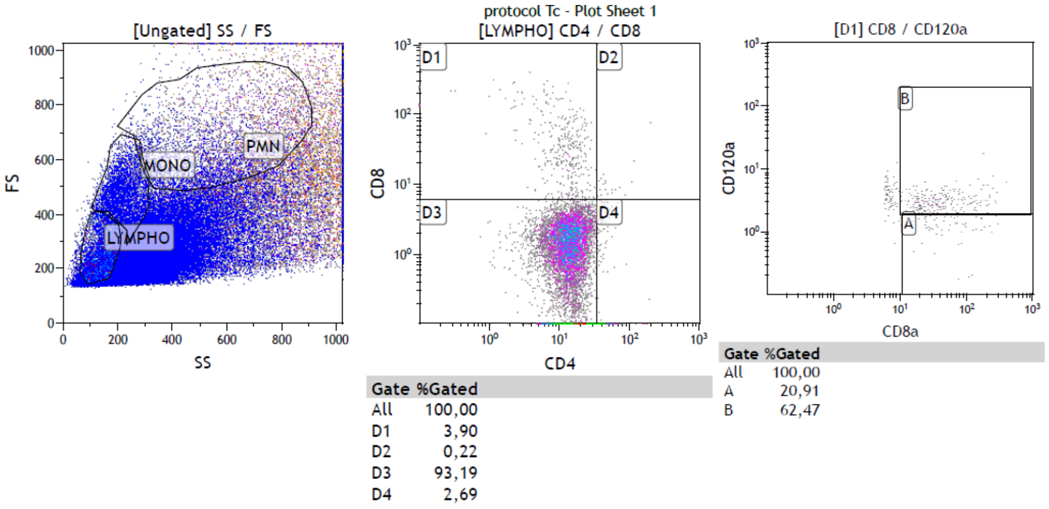

T regulator lymphocytes

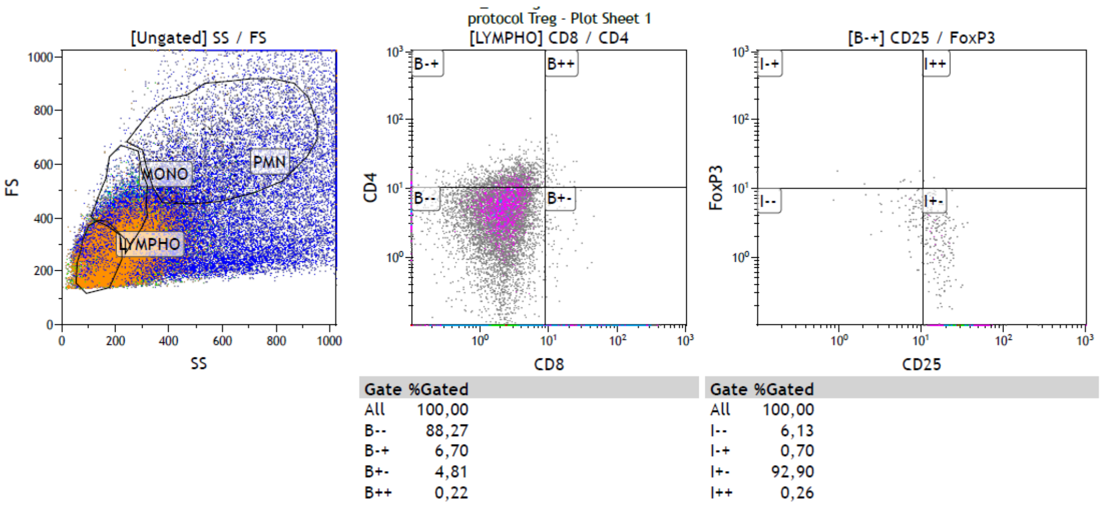

T helper 1 lymphocytes

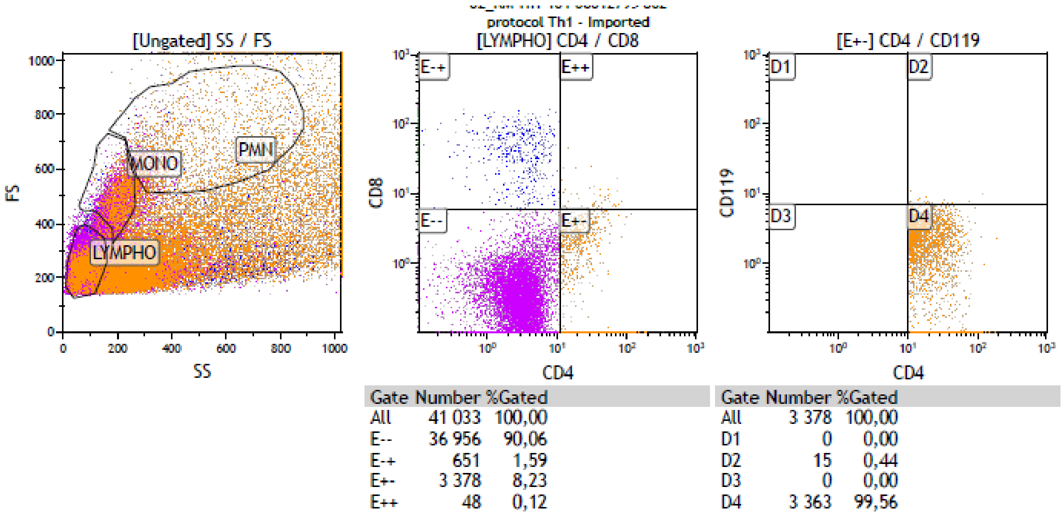

A: T helper 2 lymphocytes

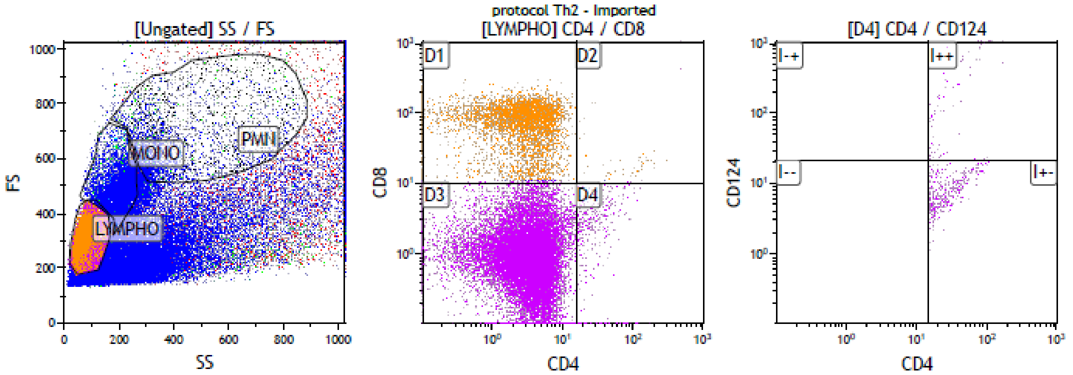

Gate	Number	%Gated
All	37 091	100,00
D1	6 755	18,21
D2	54	0,15
D3	29 846	80,47
D4	436	1,18

Gate	Number	%Gated
All	436	100,00
I--	0	0,00
I-+	0	0,00
I+-	368	84,40
I++	68	15,60

B: Natural killer cells

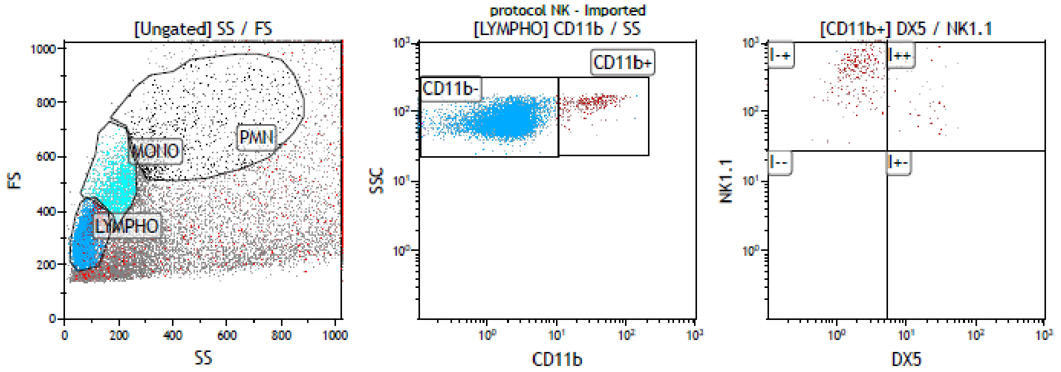

Gate	%Gated
All	100,00
CD11b-	94,43
CD11b+	3,86

Gate	%Gated
All	100,00
I--	0,00
I-+	84,87
I+-	0,00
I++	15,13

C: Natural killer T cells

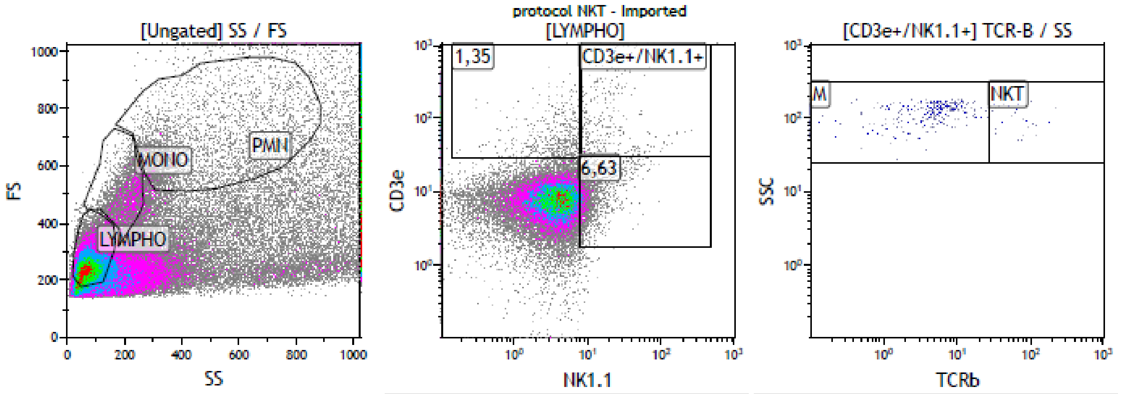

Gate	%Gated
All	100,00
1,35	1,35
6,63	6,63
CD3e+/NK1.1+	0,99

Gate	%Gated
All	100,00
M	92,53
NKT	7,47

D: Myeloid derivated suppressive cells

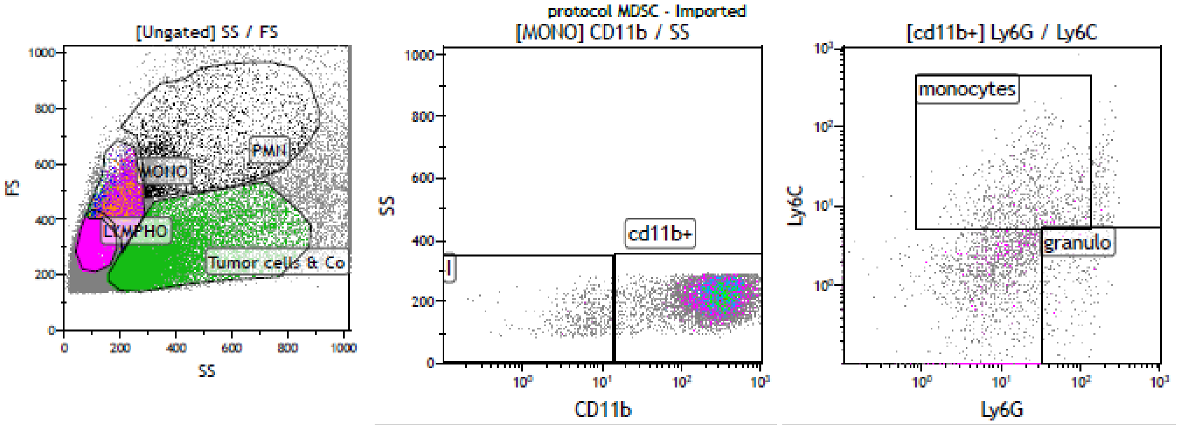

Gate	%Gated
All	100,00
cd11b+	93,98
l	5,69

Gate	%Gated
All	100,00
granulo	63,28
monocytes	10,46

E: Tumour associated macrophages

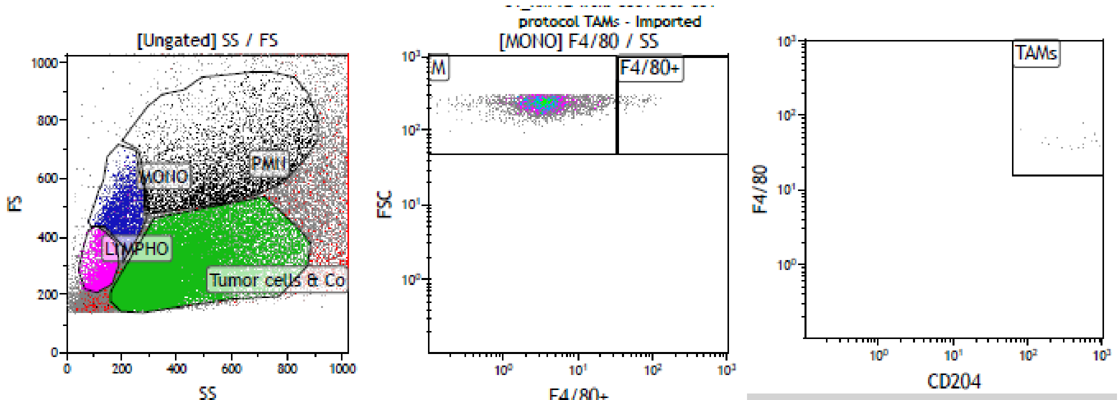

Gate	Number	%Gated
All	100 000	100,00
LYMPHO	3 873	3,87
MONO	3 907	3,91
PMN	5 459	5,46
Tumor cells & Co	53 742	53,74

Gate	%Gated
All	100,00
F4/80+	1,77
M	98,21

Gate	%Gated
All	100,00
TAMs	98,55

Figure S1. gating strategy. (**A**): T helper 2 lymphocytes, (**B**): Natural killer cells, (**C**): Natural killer T cells, (**D**): Myeloid derivated suppressive cells, (**E**): Tumour associated macrophages.
